# Pseudotargeted metabolomics revealed the adaptive mechanism of *Draba oreades* Schrenk at high altitude

**DOI:** 10.3389/fpls.2022.1052640

**Published:** 2022-12-08

**Authors:** Ling Lei, Xuefeng Yuan, Keyi Fu, Yuan Chen, Yijun Lu, Na Shou, Dandan Wu, Xi Chen, Jian Shi, Minjuan Zhang, Zhe Chen, Zunji Shi

**Affiliations:** ^1^ Clinical Psychology, Maternal and Child Health Hospital of Guangxi Zhuang Autonomous Region, Guangxi Key Laboratory of Reproductive Health and Birth Defect Prevention, Nanning, China; ^2^ State Key Laboratory of Herbage Improvement and Grassland Agro-ecosystems, Center for Grassland Microbiome, College of Pastoral Agriculture Science and Technology, Lanzhou University, Lanzhou, China; ^3^ Metabolomics Detection Department, Wuhan Metware Biotechnology Co., Ltd, Wuhan, China; ^4^ Academy of Plateau Science and Sustainability, Qinghai Normal University, Xining, China

**Keywords:** *Draba oreades* Schrenk, flavonoids, high altitude, machine learning, pseudotargeted metabolomics

## Abstract

Strong ultraviolet radiation and low temperature environment on Gangshika Mountain, located in the eastern part of the Qilian Mountains in Qinghai Province, can force plants to produce some special secondary metabolites for resisting severe environmental stress. However, the adaptive mechanism of *Draba oreades* Schrenk at high altitude are still unclear. In the current study, *Draba oreades* Schrenk from the Gangshika Mountain at altitudes of 3800 m, 4000 m and 4200 m were collected for comprehensive metabolic evaluation using pseudotargeted metabolomics method. Through KEGG pathway enrichment analysis, we found that phenylpropanoid biosynthesis, phenylalanine, tyrosine and tryptophan biosynthesis and phenylalanine metabolism related to the biosynthesis of flavonoids were up-regulated in the high-altitude group, which may enhance the environmental adaptability to strong ultraviolet intensity and low temperature stress in high altitude areas. By TopFc20 distribution diagram, the content of flavonoids gradually increased with the elevation of altitude, mainly including apigenin, luteolin, quercetin, hesperidin, kaempferol and their derivatives. Based on the random forest model, 10 important metabolites were identified as potential biomarkers. L-phenylalanine, L-histidine, naringenin-7-O-Rutinoside-4’-O-glucoside and apigenin related to the flavonoids biosynthesis and plant disease resistance were increased with the elevation of altitude. This study provided important insights for the adaptive mechanism of *Draba oreades* Schrenk at high altitude by pseudotargeted metabolomics.

## Introduction

Many commercially available drugs are obtained directly or indirectly from plant secondary metabolites (Adhikari et al., 2020). The protection and sustainable utilization of medicinal plants are crucial to the development of the traditional Chinese medicine industry, and most of China’s medicinal plants come from wild plant resources (Shan et al., 2022). The efficacy of medicinal plants mainly depends on natural secondary metabolites. In the past century, the research on natural secondary metabolites has achieved some important results, such as artemisinin for malaria, huperzine A for Alzheimer’s disease, ephedrine for cold and camptothecin for cancer, which were separated from *Artemisia annua*, *Huperzine serrata*, *Ephedra sinica* and *Camptotheca acuminate*, respectively (Qiu, 2007). However, natural secondary metabolites are susceptible to environmental factors (Setyawati et al., 2021), and the types and quantities of natural secondary metabolites vary with geographical location and altitude (Jugran et al., 2016; Cirak et al., 2017; Senica et al., 2017; Dong et al., 2020). Studies have shown that the antioxidant capacity of natural secondary metabolites are related to altitude (Ni et al., 2013; Knuesting et al., 2018; Pandey et al., 2018). Ultraviolet radiation and low temperature conditions promote the biosynthesis of flavonoids, resulting in the accumulation of flavonoids in plants (Liu et al., 2018; Dong et al., 2020).

In terms of adaptation strategies for other plants to different altitudes, Rius et al. found that the local varieties of maize adapted to high altitude in the Andes Mountains accumulated flavonoids in their leaves and other green tissues under ultraviolet radiation (Rius et al., 2012). Berardi et al. found that flavonoids were increased with elevated altitude in *Silene vulgaris* (Berardi et al., 2016). Du et al. found that the flavonoid content was higher and the flavonoid composition was markedly different in the leaves of *Cyclocarya paliurus* collected at high altitude (Du et al., 2021). Here, *Draba oreades* Schrenk is a perennial herb with wide adaptability. It mainly grows at the edge of high mountain rocks and cracks at the edge of high mountain gravel ditches with an altitude of 2300-5500 meters. It is distributed in Inner Mongolia, Shaanxi, Gansu, Qinghai, Xinjiang and other places in China, and in Central Asia, Kashmir, India and other places abroad. Up to now, the effect of altitude on the synthesis of natural secondary metabolites in *Draba oreades* Schrenk remains undetermined.

In view of this, based on liquid chromatography-mass spectrometry (LC-MS/MS) platform and sequencing platform, this study detected the shoot tissues of *Draba oreades* Schrenk at different altitudes by pseudotargeted metabolomics method, compared and analyzed the metabolome differences in the composition and quality of natural secondary metabolites of *Draba oreades* Schrenk at different altitudes, and clarified the effects of different altitudes on the accumulation of natural secondary metabolites and related regulatory mechanisms. The results of this study can provide scientific basis for the adaptive mechanism and the sustainable utilization of *Draba oreades* Schrenk at high altitude.

## Materials and methods

### Site description

Plant samples were collected from Gangshika Mountain (37°41’51.23”N~ 37°40’58.03”N, 101°28’8.11”E~101°26’49.11”E, 3800 m~4200 m a.s.l.), located in the eastern part of the Qilian Mountains in Qinghai Province. The area had a typical plateau continental climate, with a long cold winter and short warm summer. The annual average temperature was -1.6°C, with the maximum monthly mean temperature in July (10.1°C) and the minimum monthly mean temperature in January (-15.0°C). The historical extreme maximum and minimum temperatures were 26.8°C and -37.1°C, respectively. The number of days with a daily minimum temperature below 0°C during the year was as high as 280 days. The annual precipitation was 560 mm on average, of which 85% was concentrated in May to September. The regional annual mean evaporation was 1238.0 mm (Dai et al., 2019). The vegetation type of the sampling area was alpine screes cushion vegetation, and the dominant species was Thylacospermum caespitosum (Cambess.) Schischk. It was associated with Saussurea, Potentilla, Leontopodium, Gentian, Saxifraga, Poa, Oxytropis, and Polygonum. The average height of vegetation was less than 10 cm, and the aboveground dry biomass averages 210 g·m^-2^. The soil was a part of transition zone of seasonal permafrost and spot permafrost. The topsoil (0-10 cm) organic matter, bulk density, pH and soil volume water content in K. humilis meadow were 138.52 ± 13.82 g·kg^-1^, 0.75 ± 0.05 g·cm^-3^, 7.50 ± 0.22 and 32.7% ± 5.17%, respectively (Li et al., 2013). The site froze from late October to mid-November. A stable thin permafrost layer began to form in late November, and the thickness of the permafrost continued to increase, and reached the maximum freezing depth of 0.5-1 m in mid-February of the following year. The soil began to enter the thawing period in early March, and the thaw depth continued to increase until thawing was complete by late April (Dai et al., 2019).

### Sample collection

In this study, *Draba oreades* Schrenk was used as the experimental material. Samples were collected from three altitude gradients, 3800 m, 4000 m and 4200 m respectively. Eight complete plants were randomly selected at each altitude, and a total of 24 plant samples were collected. The samples were washed with distilled water immediately after collection, then frozen with liquid nitrogen, and then brought back to the laboratory for storage at - 80°C until further use. For the metabolome analysis, eight plants selected from each of the three altitude gradients were used for metabolome detection, separately.

### Metabolites extraction and detection

Metabolomics analysis of metabolites was conducted by pseudotargeted metabolomics, which can monitor hundreds to thousands of metabolites by dynamic multiple reaction monitoring, merging the advantages of untargeted and targeted metabolomics with high sensitivity, high specificity and excellent quantification ability. The standards were used to confirm the chemical identities of metabolites, which were based on the self-built target standard database MWDB (metware database) by widely target UPLC-MS/MS platform. Qualitative analysis was carried out according to the retention time RT, the information of the precursor ion pair and the secondary spectrum data of the detected metabolites. The quantification was more accurate and the repeatability was better.

The shoots of *Draba oreades* Schrenk were extracted overnight at 4°C with 70% aqueous methanol. Following centrifugation at 10000 g for 10 min, the extracts of *Draba oreades* Schrenk were absorbed (CNWBOND Carbon-GCB SPE Cartridge, 250 mg, 3 ml; ANPEL Laboratory Technologies Co., Ltd, Shanghai, China) and filtrated (SCAA-104, 0.22 μm pore size; ANPEL Laboratory Technologies Co., Ltd, Shanghai, China) before LC-ESI-MS/MS based pseudotargeted metabolomics analysis. The sample extracts were analyzed using an LC-ESI-MS/MS system (HPLC, Shim-pack UFLC SHIMADZU CBM30A system, Kyoto, Japan; MS, Applied Biosystems 4500 QTRAP, Foster City, California, USA). The analytical conditions were as follows: column, Waters ACQUITY UPLC HSS T3 C18 (1.8 µm, 2.1 mm*100 mm); solvent system, water (0.04% acetic acid): acetonitrile (0.04% acetic acid); gradient program,100: 0 V/V at 0 min, 5: 95 V/V at 11.0 min, 5: 95 V/V at 12.0 min, 95: 5 V/V at 12.1 min, 95: 5 V/V at 15.0 min; flow rate, 0.40 ml/min; temperature, 40°C; injection volume: 5μl. The effluent was connected to an ESI-triple quadrupole-linear ion trap (QTRAP)-MS (Applied Biosystems Co., Ltd, Foster City, California, USA). Triple quadrupole (QQQ) scans were acquired on a triple quadrupole-linear ion trap mass spectrometer (QTRAP). The ESI source operation parameters were as follows: ion source, turbo spray; source temperature 550°C; ion spray voltage (IS) 5500 V; ion source gas I (GSI), gas II (GSII), and curtain gas (CUR) were set at 55, 60, and 25.0 psi, respectively; the collision gas (CAD) was high. Instrument tuning and mass calibration were performed with 10 μmol/l polypropylene glycol solutions in QQQ modes. QQQ scans were acquired as MRM experiments with collision gas (i.e., nitrogen) set to 5 psi. DP and CE for individual MRM transitions were done with further DP and CE optimization. A specific set of MRM transitions was monitored for each period according to the metabolites eluted within this period.

### Metabolomics data analysis

For metabolomics data analysis, the metabolomics data obtained by LC-ESI-MS/MS system were normalized with log_2_ transformation and standard with z-score, which were then exported into SIMCA 14.1 software to analyze the differential metabolites of samples. Quality control (QC) samples were a mixture of all sample extracts, which were inserted into the queue to monitor the stability of the detection method. Unsupervised principal component analysis (PCA) was performed to estimate the degree of variability and the overall metabolic difference. The metabolomics data were analyzed according to the orthogonal partial least structures discriminant analysis (OPLS-DA) model. The scores of each group were plotted to display the differences between each group. A combination of Fold change (FC) values, *P*-values, and Variable important in projection (VIP) values from the OPLS-DA model can be used to screen for differential metabolites, and the validity of OPLS-DA models was further confirmed by permutation tests. Volcano plot and Venn diagram were used to illustrate the number of differential metabolites. The pathway enrichment analysis of differential metabolites was conducted based on the Kyoto Encyclopedia of genes and genomes (KEGG) pathway database (http://www.kegg.jp/kegg/pathway.html) (Kanehisa and Goto, 2000).

### Statistical analysis

We used R Programming Language (R 4.1.0, New Zealand) and GraphPad software (8.0 version, United States) for graphic illustration and statistical analysis. The differential metabolites between groups were screened by VIP values, *P*-values, and FC values (VIP > 1, *P* < 0.05, FC ≥ 2 or ≤ 0.5). One-way ANOVA analysis or two-tailed Student’s t test were conducted for calculating the significant difference between the two groups.

## Results

### Characterization of soil samples and measurement of total ultraviolet intensity

The soil samples and plant samples were collected from the Gangshika Mountain at altitudes of 3800 m (E 101°26’49.11”, N 37°40′58.03”), 4000 m (E 101°27’4.56”, N 37°41′40.05”) and 4200 m (E 101°28’8.11”, N 37°41′51.23”). The physicochemical properties of the soil samples were presented in **Table S1**. In the low-altitude group, the average water content was 39.5%, the average salinity was 50.5 mg/L, and the average conductivity was 92.4 us/cm, all which were higher than the mid-altitude and high-altitude groups. Notably, the average soil temperature at 4200 m was 1.9°C, which was lower than the average soil temperature at 4000 m and at 3800 m. Moreover, total ultraviolet intensity of plant growing season from May to September showed the increasing trend with the elevation of altitudes (**Table S2**).

### Quality control analysis of metabolomics data

**Figure 1:**
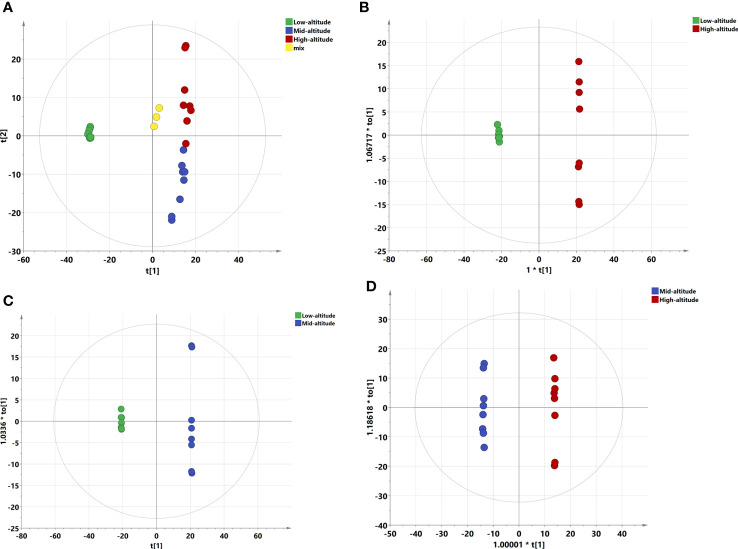
PCA and OPLS-DA scoring charts of *Draba oreades* Schrenk at different altitudes. **(A)** PCA plot. **(B)** OPLS-DA model score at Low-altitude vs High-altitude. **(C)** OPLS-DA model score at Low-altitude vs Mid-altitude. **(D)** OPLS-DA model score at Mid-altitude vs High-altitude. QC, quality control.

QC samples were used to determine the state of the instrument and balance the LC-MS system to evaluate the stability of the system during the whole experiment. Through the QC analysis of the original LC-MS data, the variation coefficient distribution of the three groups of samples and QC samples were obtained. The abscissa represented the CV value, the ordinate represented the proportion of the number of metabolites smaller than the corresponding CV value to the total number of metabolites. The higher the proportion of metabolites with lower CV values in QC samples, the more stable the experimental data was. The proportion of metabolites with CV value less than 0.3 in QC samples (mix samples were used for QC) was higher than 85%, indicating the reliability of the measurement technology (**Figure S1A**). The stability of the determination method was evaluated by superimposing the total ion current (TIC) of the four groups of samples, the ordinate represented the peak intensity and the abscissa represented the retention time. The results showed that the TIC in the positive and negative ion mode were highly overlapped, the retention time was consistent with the peak intensity, indicating that the peak separation effect was good, and the signal was stable in the whole analysis process (**Figures S1B, C**). In addition, a total of 776 metabolites were detected by UHPLC-TQMS using hydrophilic and hydrophobic methods. PCA results of all metabolites showed that QC samples gathered together, further indicating the stability of the analytical method (**Figure 1A**).

### Metabolomic analysis

PCA analysis can reflect the distribution of relative abundance of metabolites. The closer the distance was, the smaller the difference of substance content was. In this study, PCA results of three groups of samples and QC samples were all within the confidence interval, there were significant differences among groups (*P*=0.035), but no obvious separation of samples in the same group, indicating that the representativeness was good (**Figure 1A**). PCA was an unsupervised analysis method, which cannot ignore intra group errors and eliminate random errors irrelevant to the research purpose, so it was not conducive to the discovery of inter group differences. After that, the supervised method will be used for further research.

Through the supervised method OPLS-DA, the orthogonal variables that were not related to the classification variables in metabolites can be screened out, and the nonorthogonal variables and orthogonal variables can be analyzed respectively, so as to obtain more reliable information about the correlation between the inter group differences of metabolites and the experimental group. The low altitude samples in OPLS-DA model were mainly distributed on the left side of the confidence interval, and the high altitude samples were mainly distributed on the right side of the confidence interval. The separation effect of the two altitude samples was significant, R^2^X=0.807, Q^2 =^ 0.999, and the two parameter values were greater than 0.5, indicating that the model was reliable (**Figure 1B**). The OPLS-DA model had a significant separation between the low-altitude group and the mid-altitude group, R^2^X=0.777, Q^2 =^ 0.999 (**Figure 1C**). The mid-altitude group and the high-altitude group had also a significant separation, R^2^X=0.641, Q^2 =^ 0.975 (**Figure 1D**). Moreover, *P*-values of CV-ANOVA of OPLS-DA models in pairwise comparisons were all less than 0.001, further indicating that there were significant differences among the three groups.

OPLS-DA permutation test can obtain R^2^ and Q^2^ values of the random model by randomly changing the arrangement order of classification variables Y and establishing the corresponding OPLS-DA model for many times. It played an important role in avoiding over fitting of the test model and evaluating the statistical significance of the model. Our results showed that all R^2^ points of the three groups of samples were lower than the rightmost original R^2^ point from left to right, and all Q^2^ points were lower than the original Q^2^ point on the right. Moreover, the regression line and ordinate of the points intersected the negative semi axis, indicating that the OPLS-DA model was reliable and robust without over fitting, which can better explain the difference between the two groups (**Figure S2**).

### Screening of differential metabolites

**Figure 2:**
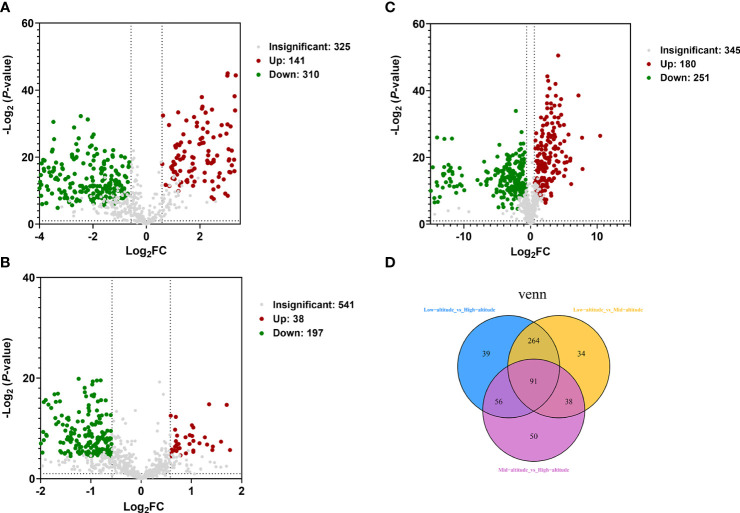
Volcano plot and Venn diagram were used to illustrate the number of differential metabolites. **(A)** Volcano plot of Low-altitude vs High-altitude. **(B)** Volcano plot of Low-altitude vs Mid-altitude. **(C)** Volcano plot of Mid-altitude vs High-altitude. **(D)** Venn diagram showed the common differential metabolites and unique differential metabolites at different altitudes.

The differential metabolites of *Draba oreades* Schrenk at different altitudes were screened through the volcano plot. The abscissa represented the logarithm of the difference multiple of the differential metabolites [log_2_(FC)], and the ordinate represented the negative logarithm of *P*-value [- log _10_ (*P*-value)]. The metabolites at low, middle and high altitudes were compared in pairs. In the comparison group of low-altitude vs high-altitude, there were 451 differential metabolites, 141 up-regulated metabolites and 310 down-regulated metabolites (**Figure 2A**). In the comparison group of low-altitude vs mid-altitude, there were 431 differential metabolites, 180 up-regulated metabolites and 251 down-regulated metabolites (**Figure 2B**). In the comparison group of mid-altitude vs high-altitude, there were 235 differential metabolites, 38 up-regulated metabolites and 197 down-regulated metabolites (**Figure 2C**). Meanwhile, we found that the number of differential metabolites was the largest in the mid-altitude vs high-altitude comparison group, and the least in the low-altitude vs high-altitude comparison group, and the down-regulated differential metabolites were far more than the up-regulated differential metabolites in the three comparison groups. Venn analysis was carried out on the differential metabolites of *Draba oreades* Schrenk at different altitudes, 91 common differential metabolites were obtained by independent pairwise comparison. There were 39, 34 and 50 unique metabolites in the comparison of low-altitude vs high-altitude, low-altitude vs mid-altitude, mid-altitude vs high-altitude respectively (**Figure 2D**).

### Analysis of metabolic pathways of differential metabolites

**Figure 3:**
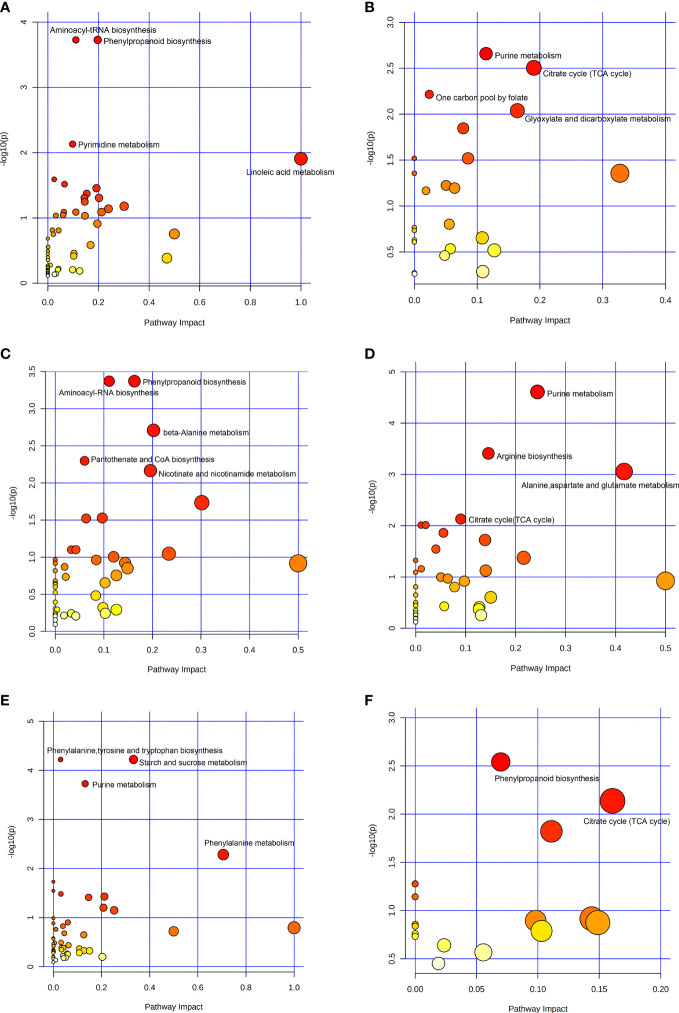
Bubble plot of KEGG pathway enrichment analysis. **(A)** Metabolic pathways of up-regulated differential metabolites at Low-altitude vs High-altitude. **(B)** Metabolic pathways of down-regulated differential metabolites at Low-altitude vs High-altitude. **(C)** Metabolic pathways of up-regulated differential metabolites at Low-altitude vs Mid-altitude. **(D)** Metabolic pathways of down-regulated differential metabolites at Low-altitude vs Mid-altitude. **(E)** Metabolic pathways of up-regulated differential metabolites at Mid-altitude vs High-altitude. **(F)** Metabolic pathways of down-regulated differential metabolites at Mid-altitude vs High-altitude.

KEGG pathway enrichment analysis was performed for the up-regulated and down-regulated differential metabolites screened in the volcano plot, as shown in **Figure 3**. The ordinate was - log_10_ (*P*), and the abscissa was pathway impact. The size of the dot represented the number of differential metabolites enriched in the corresponding pathway, and the color of the dot represented the *P* value. The smaller the *P* value, the redder the color of the dot. The metabolic pathways of the up-regulated differential metabolites between the low-altitude and the high-altitude were mainly aminoacyl-tRNA biosynthesis, phenylpropanoid biosynthesis, pyrimidine metabolism and linoleic acid metabolism (**Figure 3A**), the metabolic pathways of the down-regulated differential metabolites were mainly purine metabolism, citrate cycle, one carbon pool by folate, and glyoxylate and dicarboxylate metabolism (**Figure 3B**). The metabolic pathways of the up-regulated differential metabolites between the low-altitude and the mid-altitude were mainly phenylpropanoid biosynthesis, aminoacyl-tRNA biosynthesis, beta-Alanine metabolism, pantothenate and CoA biosynthesis, and nicotinate and nicotinamide metabolism (**Figure 3C**), the metabolic pathways of the down-regulated differential metabolites were mainly purine metabolism, arginine biosynthesis, alanine, aspartate and glutamate metabolism and citrate cycle (**Figure 3D**). The metabolic pathways of the up-regulated differential metabolites between the mid-altitude and the high-altitude were mainly phenylalanine, tyrosine and tryptophan biosynthesis, starch and sucrose metabolism, purine metabolism and phenylalanine metabolism (**Figure 3E**), the metabolic pathways of the down-regulated differential metabolites were mainly phenylpropanoid biosynthesis and citrate cycle (**Figure 3F**).

### Analysis of the top 20 changed differential metabolites

**Figure 4:**
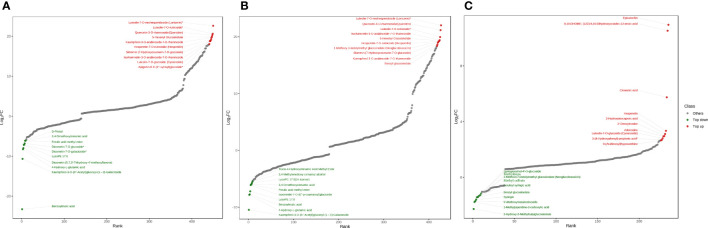
TopFc20 distribution diagram. **(A)** Low-altitude vs High-altitude. **(B)** Low-altitude vs Mid-altitude. **(C)** Mid-altitude vs High-altitude.

In order to further understand the different metabolites with great differences in the comparison groups at different altitudes, the topFc20 distribution map screened the compounds with the top 20 changes in the relative content of different metabolites. Red represented the 10 compounds with increased relative content, and green represented the 10 compounds with decreased relative content. In the low-altitude and high-altitude comparison group, the flavonoids including luteolin-7-O-neohesperidoside (lonicerin), luteolin-7-O-rutinoside, hesperetin-7-O-rutin (hesperidin), kaempferol-3-O-arabinoside-7-O-rhamnoside, hesperetin-7-O-rutinoside (hesperidin), isorhamnetin-3-O-arabinoside-7-O-rhamnoside, luteolin-7-O-glucoside (cynaroside), apigenin-6-C-(2”-xylosyl) glucoside and the coumarin including 7-Hydroxycoumarin-7-O-glucoside (skimmin) were up-regulated at high altitude, which were important pharmacological compounds (**Figure 4A**). In the low-altitude and mid-altitude comparison group, the flavonoids including lonicerin, hesperidin, luteolin-7-O-rutinoside, isorhamnetin-3-O-arabinoside-7-O-rhamnoside, kaempferol-3-O-arabinoside-7-O-rhamnoside and the coumarin including skimmin were up-regulated in mid-altitude (**Figure 4B**). In the mid-altitude and high-altitude comparison group, the flavonoids including epicatechin, hesperetin, cynaroside and the organic acid including cinnamic acid were up-regulated at high altitude (**Figure 4C**).

### Machine learning and receiver operating characteristic curve analysis

**Figure 5:**
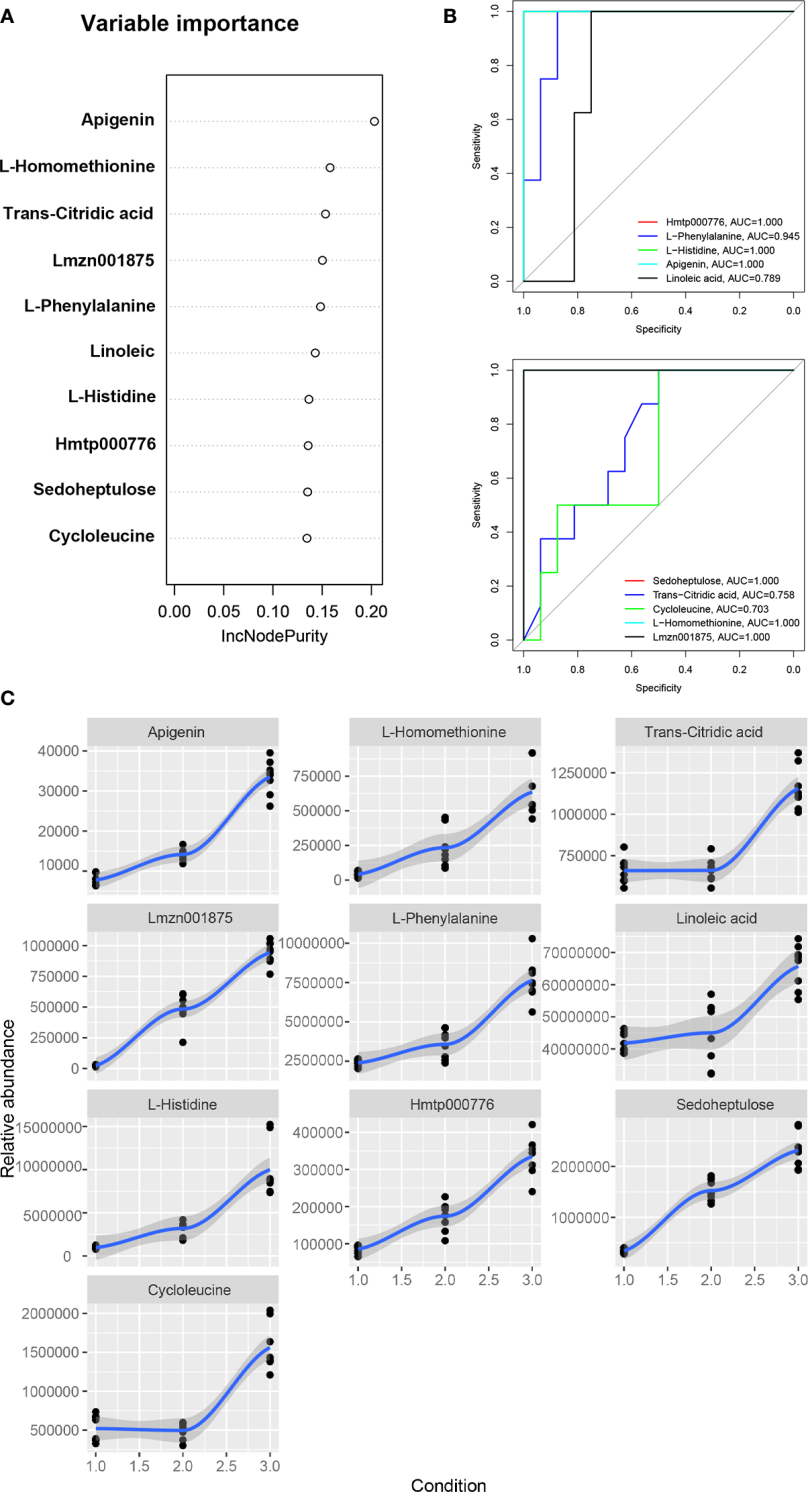
Machine learning and receiver operating characteristic curve analysis. **(A)** The random forest model was used to screen 10 metabolic biomarkers for *Draba oreades* Schrenk at different altitudes. **(B)** ROC curves of biomarkers. **(C)** Relative abundance trend charts of 10 biomarkers at different altitudes. The abscissa 1.0 represented low-altitude, 2.0 represented mid-altitude, and 3.0 represented high-altitude. Lmzn001875: Naringenin-7-O-Rutinoside-4’-O-glucoside, Hmtp000776: 4,5,6-Trihydroxy-2-cyclohexen-1-ylideneacetonitrile.

The machine learning method of random forest model was used to screen the biomarkers for the metabolites of *Draba oreades* Schrenk at different altitudes. In the random forest model, 10 important metabolites were separated as potential biomarkers according to the relative importance of metabolites (**Figure 5A**). The abscissa of the Receiver operating characteristic (ROC) curve of biomarkers represents specificity, the ordinate represents sensitivity, and the curves of different colors represented different metabolites. The area enclosed by the curve and the abscissa was a value called area under the curve (AUC) (**Figure 5B**). AUC value greater than 0.6 indicated that the machine learning model was effective. The higher the AUC value, the better the predictability of biomarkers. In the current study, Hmtp000776 (4,5,6-Trihydroxy-2-cyclohexen-1-ylideneacetonitrile), Lmzn001875 (naringenin-7-O-Rutinoside-4’-O-glucoside), L-phenylalanine, L-histidine, apigenin, linoleic acid, sedoheptulose, cycloleucine, trans-citridic acid and L-homomethionine were identified as potential biomarkers of *Draba oreades* Schrenk at high altitude (**Figure 5A**). The AUC values of these 10 potential biomarkers were all greater than 0.6, indicating that these metabolites were biomarkers to distinguish between different altitudes (**Figure 5B**). And the results showed that the relative abundance of 10 biomarkers increased with the increase of altitude (**Figure 5C**).

## Discussions

Previous studies have shown that natural secondary metabolites of plants have multiple functions (Shang et al., 2021). However, due to the limitations of natural resources or low contents in plants, the supply of natural secondary metabolites is often curtailed and the components of natural secondary metabolites remain unclear (Yang et al., 2016). Previous study found that the content of Salidroside in *Rhodiola sachalinensis* was related to the altitudes (Zhao et al., 2014; Dong et al., 2020), suggesting that altitude may be an important factor that affects phytochemicals. Thus, we need to study the impact of environmental factors on plant secondary metabolites, which will help to determine the favorable geographical location for the plants with important value (Adhikari et al., 2020). However, so far, the effect of altitude on the synthesis of natural secondary metabolites in *Draba oreades* Schrenk remains undetermined, suggesting that it may be of great significance to systematically analyze and screen the metabolites of *Draba oreades* Schrenk under different altitudes. In this study, based on the pseudotargeted metabolomics analysis of LC-MS, the global metabolites of *Draba oreades* Schrenk from three different altitudes were determined. The effects of altitude on the metabolites of *Draba oreades* Schrenk were discussed.

The samples were subjected to multivariate analysis such as PCA and OPLS-DA by pseudotargeted metabolomics, and 776 metabolites were identified in three different altitudes of *Draba oreades* Schrenk. The differential metabolites of *Draba oreades* Schrenk among low, middle or high altitudes were also compared. The results showed that the differential metabolites could be used to distinguish the samples from different altitudes, indicating that the species and relative abundance of metabolites in different altitudes were different, and the altitude had a significant effect on the metabolism of *Draba oreades* Schrenk. Farhat et al. found that the changes of altitude would lead to significant changes in phenolic content and antioxidant activity of *Salvia verbenaca L.* (Farhat et al., 2013). Setyawati et al. found that altitude had an impact on the content of secondary metabolites of turmeric and ginger, the content of secondary metabolites of turmeric and ginger was higher in high altitude areas and decreased with the decrease of altitude (Setyawati et al., 2021).

KEGG pathway enrichment analysis showed that the metabolic pathways of significant enrichment in *Draba oreades* Schrenk at different altitudes. Previous studies showed that phenylpropanoid biosynthesis was crucial to the development and survival of plants, it produced precursor metabolites with multiple functions, which can help plants cope with non-biological and biological stresses in the environment. Among the precursor metabolites, the most significant compounds were flavonoids which were related to UV protection and low temperature resistance, and other compounds were involved in the plant’s defense response to pathogens (De Vries et al., 2021; Dong and Lin, 2021). Dong et al. found that the phenylpropanoid biosynthesis promoted the synthesis of flavonoids with the increase of altitude in *Rhodiola* (Dong et al., 2020). Aminoacyl-tRNA biosynthesis played an important role in protein synthesis (Jiang et al., 2020). Phenylalanine, tyrosine and tryptophan biosynthesis was involved in the synthesis of three aromatic amino acids including tryptophan, tyrosine and phenylalanine, tryptophan was the key precursor of the IAA biosynthetic pathway in plants (Zhao, 2012), tyrosine may help to improve the drought tolerance of plants (Ma et al., 2017), and phenylalanine was the key precursor of flavonoids production in plants (Meng et al., 2019). Beta-Alanine metabolism was involved in the synthesis of pantothenic acid, and beta-Alanine was methylated to beta-Alanine betaine in stress resistant plants which was an osmotic protective compound in response to high salt and hypoxia (Rathinasabapathi et al., 2001). Starch and sucrose metabolism was related to the tolerance and sensitivity of plants to abiotic stresses (Dev Sharma et al., 2021). In this study, we found that phenylpropanoid biosynthesis and aminoacyl-tRNA biosynthesis were up-regulated in the mid-altitude and high-altitude compared to the low-altitude. Phenylalanine, tyrosine and tryptophan biosynthesis, starch and sucrose metabolism and phenylalanine metabolism were up-regulated in the high-altitude compared to the mid-altitude. Beta-Alanine metabolism was up-regulated in the mid-altitude compared to the low-altitude. Collectively, *Draba oreades* Schrenk may enhance the biosynthesis of flavonoids through above important metabolic pathways to adapt to the environmental challenges brought by high altitude.

Purine metabolism retransported nitrogen for plant growth and development (Watanabe et al., 2014). Citrate cycle was a key pathway for cells to generate energy during metabolism and provide precursors for biosynthetic reaction, closely relating to plant growth (Zhang et al., 2018; Arnold et al., 2022). In this study, it can be seen that these metabolic pathways related to plant growth and development were down-regulated in the mid-altitude and high-altitude compared to the low-altitude, such as purine metabolism and citrate cycle, duo to the harsh high altitude environment. In addition, glyoxylate and dicarboxylate metabolism, pyrimidine metabolism, one carbon pool by folate, arginine metabolism, and linoleic acid metabolism showed the unstable trend at different altitudes.

TopFc20 distribution diagram screened the up-regulated differential metabolites in the three comparison groups of low, middle and high altitude, mainly including flavonoids with important pharmacological activities such as luteolin-7-O-neohesperidoside (lonicerin), luteolin-7-O-rutinoside, hesperetin-7-O-rutin (hesperidin), kaempferol-3-O-arabinoside-7-O-rhamnoside, hesperetin-7-O-rutinoside (hesperidin), isorhamnetin-3-O-arabinoside-7-O-rhamnoside, luteolin-7-O-glucoside (cynaroside), apigenin-6-C-(2”-xylosyl) glucoside, epicatechin, hesperetin, including organic acids such as cinnamic acid, and including coumarin components such as skimmin. In this study, with the increase of altitude, the more flavonoids including luteolin, quercetin, apigenin and their derivatives were accumulated. It was reported that luteolin had anti-tumor effects on a variety of human malignant tumors, such as lung cancer, breast cancer, glioblastoma, prostate cancer, colon cancer and pancreatic cancer (Imran et al., 2019). Quercetin was an antioxidant flavonoid widely distributed in plants and was a promising anticancer agent (Murakami et al., 2008; Li and Song, 2019). Apigenin had preventive and therapeutic effects on cardiovascular diseases and nervous system diseases (Shukla and Gupta, 2010). In addition, skimmin (7-Hydroxycoumarin-7-O-glucoside) had preventive effects on diabetes and nephropathy (Zhang et al., 2012; Zhang et al., 2013). Studies showed that ultraviolet radiation changed the secondary metabolism of plants, kaempferol had important protective effects against ultraviolet damage (Tripp et al., 2018). The concentrations of two flavonoids with the highest absorbance in the ultraviolet wavelength, apigenin and luteon, were positively correlated with high latitude areas (Tripp et al., 2018). This explained why the content of flavonoids in plant metabolites in high altitude areas was higher than that in low altitude areas.

Based on the random forest model, 10 important metabolites were identified as potential biomarkers of *Draba oreades* Schrenk in this study, and the relative abundance of the 10 biomarkers increased with the elevation of altitude. Among them, Lmzn001875 (naringenin-7-O-Rutinoside-4’-O-glucoside), Hmtp000776 (4,5,6-Trihydroxy-2-cyclohexen-1-ylideneacetonitrile), L-phenylalanine, L-histidine, apigenin, linoleic acid were the main biomarkers with pharmacological activities in SymMap database. Linoleic acid was the most abundant polyunsaturated fatty acid in human nutrition (Choque et al., 2014; Imran et al., 2019). Amino acids including L-phenylalanine and L-histidine, as substrates of protein biosynthesis, played a regulatory role in this process (Heinemann and Hildebrandt, 2021). Phenylalanine can be used as the starting substrate of flavonoid biosynthesis, which played a variety of functions in plants (Cheng et al., 2014). L-histidine can induce plants to enhance resistance to plant diseases (Yariyama et al., 2019). Some studies showed that the flavonoid content in plants was significantly positively correlated with altitude (Adhikari et al., 2020; Dong et al., 2020), which was correlated with the stronger ultraviolet radiation and low temperature stress in high altitude areas (Hectors et al., 2012; Emiliani et al., 2013). Under the stimulation of strong light, the most effective protective mechanism was the biosynthesis of flavonoids substances (Frohnmeyer and Staiger, 2003). In the current study, our results suggested that the more ultraviolet intensity and the lower temperature at 4200 m exhibited more severe environmental stress compared to at 4000 m and at 3800 m (**Tables S1**, **S2**). Notably, the flavonoids as potential biomarkers including naringenin-7-O-Rutinoside-4’-O-glucoside and apigenin, and the flavonoid synthesis related metabolic pathways were markedly up-regulated at 4200 m in the study.

In summary, it was found that there were significant differences in the metabolites of *Draba oreades* Schrenk at different altitudes through pseudotargeted metabolomics analysis. Phenylpropanoid biosynthesis, phenylalanine, tyrosine and tryptophan biosynthesis and phenylalanine metabolism related to the biosynthesis of flavonoids were up-regulated in the high-altitude group, which may enhance the environmental adaptability to strong ultraviolet intensity and low temperature stress in high altitude areas. Purine metabolism and citrate cycle related to plant growth and development were down-regulated in the mid-altitude and high-altitude groups, duo to the harsh high altitude environment. By TopFc20 distribution diagram, the content of flavonoids gradually increased with the elevation of altitude, mainly including apigenin, luteolin, quercetin, hesperidin, kaempferol and their derivatives. Based on the random forest model, 10 important metabolites were identified as potential biomarkers. Notably, L-phenylalanine, L-histidine, naringenin-7-O-Rutinoside-4’-O-glucoside and apigenin related to the flavonoids biosynthesis and plant disease resistance were increased with the elevation of altitude. This study provides important insights for the environment adaptability of *Draba oreades* Schrenk to high altitude.

## Data availability statement

The original contributions presented in the study are included in the article/**Supplementary Material**. Further inquiries can be directed to the corresponding authors.

## Author contributions

ZS, project administration, supervision, experimental design, validation, visualization, writing-review and editing, and funding acquisition. ZC, supervision and funding acquisition. LL, investigation, resources, methodology, and data curation. XY, data curation, visualization, and writing-original draft. KF, investigation and visualization. YC, investigation. YL, resources, visualization. NS, visualization. DW and XC, validation. JS and MZ, methodology. All authors contributed to the article and approved the submitted version.

## References

[B1] Adhikari P. Joshi K. Singh M. Pandey A. (2020). Influence of altitude on secondary metabolites, antioxidants, and antimicrobial activities of Himalayan yew (Taxus wallichiana). Plant Biosyst. 156 (1), 187–195. doi: 10.1080/11263504.2020.1845845

[B2] Arnold P. K. Jackson B. T. Paras K. I. Brunner J. S. Hart M. L. Newsom O. J.. (2022). A non-canonical tricarboxylic acid cycle underlies cellular identity. Nature 603 (7901), 477–481. doi: 10.1038/s41586-022-04475-w 35264789 PMC8934290

[B3] Berardi A. E. Fields P. D. Abbate J. L. Taylor D. R. (2016). Elevational divergence and clinal variation in floral color and leaf chemistry in silene vulgaris. Am. J. Bot. 103 (8), 1508–1523. doi: 10.3732/ajb.1600106 27519429

[B4] Cheng A. X. Han X. J. Wu Y. F. Lou H. X. (2014). The function and catalysis of 2-oxoglutarate-dependent oxygenases involved in plant flavonoid biosynthesis. Int. J. Mol. Sci. 15 (1), 1080–1095. doi: 10.3390/ijms15011080 24434621 PMC3907857

[B5] Choque B. Catheline D. Rioux V. Legrand P. (2014). Linoleic acid: Between doubts and certainties. Biochimie 96, 14–21. doi: 10.1016/j.biochi.2013.07.012 23900039

[B6] Cirak C. Radusiene J. Jakstas V. Ivanauskas L. Seyis F. Yayla F. (2017). Altitudinal changes in secondary metabolite contents of hypericum androsaemum and hypericum polyphyllum. Biochem. Systematics Ecol. 70, 108–115. doi: 10.1016/j.bse.2016.11.006

[B7] Dai L. C. Guo X. W. Zhang F. W. Du Y. G. Ke X. Li Y. K.. (2019). Seasonal dynamics and controls of deep soil water infiltration in the seasonally-frozen region of the qinghai-Tibet plateau. J. Hydrol. 571, 740–748. doi: 10.1016/j.jhydrol.2019.02.021

[B8] De Vries S. Furst-Jansen J. M. R. Irisarri I. Dhabalia Ashok A. Ischebeck T. Feussner K.. (2021). The evolution of the phenylpropanoid pathway entailed pronounced radiations and divergences of enzyme families. Plant J. 107 (4), 975–1002. doi: 10.1111/tpj.15387 34165823

[B9] Dev Sharma K. Patil G. Kiran A. (2021). Characterization and differential expression of sucrose and starch metabolism genes in contrasting chickpea (Cicer arietinum l.) genotypes under low temperature. J. Genet. 100, 71. doi: 10.1007/s12041-021-01317-y 34608872

[B10] Dong X. Guo Y. Xiong C. Sun L. (2020). Evaluation of two major rhodiola species and the systemic changing characteristics of metabolites of rhodiola crenulata in different altitudes by chemical methods combined with UPLC-QqQ-MS-Based metabolomics. Molecules 25 (18), 4062. doi: 10.3390/molecules25184062 32899531 PMC7570721

[B11] Dong N. Q. Lin H. X. (2021). Contribution of phenylpropanoid metabolism to plant development and plant-environment interactions. J. Integr. Plant Biol. 63 (1), 180–209. doi: 10.1111/jipb.13054 33325112

[B12] Du Z. Lin W. Yu B. Zhu J. Li J. (2021). Integrated metabolomic and transcriptomic analysis of the flavonoid accumulation in the leaves of cyclocarya paliurus at different altitudes. Front. Plant Sci. 12. doi: 10.3389/fpls.2021.794137 PMC8860981 35211131

[B13] Emiliani J. Grotewold E. Ferreyra M. L. F. Casati P. (2013). Flavonols protect arabidopsis plants against UV-b deleterious effects. Mol. Plant 6 (4), 1376–1379. doi: 10.1093/mp/sst021 23371934

[B14] Farhat M. B. Landoulsi A. Chaouch-Hamada R. Sotomayor J. A. Jordán M. J. (2013). Phytochemical composition and *in vitro* antioxidant activity of by-products of salvia verbenaca l. growing wild in different habitats. Ind. Crops Prod. 49, 373–379. doi: 10.1016/j.indcrop.2013.05.006

[B15] Frohnmeyer H. Staiger D. (2003). Ultraviolet-b radiation-mediated responses in plants. balancing damage and protection. Plant Physiol. 133 (4), 1420–1428. doi: 10.1104/pp.103.030049 14681524 PMC1540342

[B16] Hectors K. van Oevelen S. Guisez Y. Prinsen E. Jansen M. A. (2012). The phytohormone auxin is a component of the regulatory system that controls UV-mediated accumulation of flavonoids and UV-induced morphogenesis. Physiol. Plant 145 (4), 594–603. doi: 10.1111/j.1399-3054.2012.01590.x 22304327

[B17] Heinemann B. Hildebrandt T. M. (2021). The role of amino acid metabolism in signaling and metabolic adaptation to stress-induced energy deficiency in plants. J. Exp. Bot. 72 (13), 4634–4645. doi: 10.1093/jxb/erab182 33993299

[B18] Imran M. Rauf A. Abu-Izneid T. Nadeem M. Shariati M. A. Khan I. A.. (2019). Luteolin, a flavonoid, as an anticancer agent: A review. BioMed. Pharmacother. 112, 108612. doi: 10.1016/j.biopha.2019.108612 30798142

[B19] Jiang L. Jones J. Yang X. L. (2020). Human diseases linked to cytoplasmic aminoacyl-tRNA synthetases. Enzymes 48, 277–319. doi: 10.1016/bs.enz.2020.06.009 33837707

[B20] Jugran A. K. Bahukhandi A. Dhyani P. Bhatt I. D. Rawal R. S. Nandi S. K. (2016). Impact of altitudes and habitats on valerenic acid, total phenolics, flavonoids, tannins, and antioxidant activity of valeriana jatamansi. Appl. Biochem. Biotechnol. 179 (6), 911–926. doi: 10.1007/s12010-016-2039-2 26971960

[B21] Kanehisa M. Goto S. (2000). KEGG: kyoto encyclopedia of genes and genomes. Nucleic Acids Res. 28 (1), 27–30. doi: 10.1093/nar/28.1.27 10592173 PMC102409

[B22] Knuesting J. Brinkmann M. C. Silva B. Schorsch M. Bendix J. Beck E.. (2018). Who will win where and why? an ecophysiological dissection of the competition between a tropical pasture grass and the invasive weed bracken over an elevation range of 1000 m in the tropical Andes. PloS One 13 (8), e0202255. doi: 10.1371/journal.pone.0202255 30102718 PMC6089443

[B23] Li Y. Dong S. Wen L. Wang X. Wu Y. (2013). The effects of fencing on carbon stocks in the degraded alpine grasslands of the qinghai-Tibetan plateau. J. Environ. Manage 128, 393–399. doi: 10.1016/j.jenvman.2013.05.058 23792816

[B24] Li Q. Song J. (2019). Analysis of widely targeted metabolites of the euhalophyte suaeda salsa under saline conditions provides new insights into salt tolerance and nutritional value in halophytic species. BMC Plant Biol. 19 (1), 388. doi: 10.1186/s12870-019-2006-5 31492100 PMC6729093

[B25] Liu Y. Liu J. Wang Y. Abozeid A. Tian D. M. Zhang X. N.. (2018). The different resistance of two astragalus plants to UV-b stress is tightly associated with the organ-specific isoflavone metabolism. Photochem. Photobiol. 94 (1), 115–125. doi: 10.1111/php.12841 28881500

[B26] Ma J. H. Gao X. L. Liu Q. Shao Y. Zhang D. J. Jiang L. N.. (2017). Overexpression of TaWRKY146 increases drought tolerance through inducing stomatal closure in arabidopsis thaliana. Front. Plant Sci. 8. doi: 10.3389/fpls.2017.02036 PMC5706409 29225611

[B27] Meng J. Wang B. He G. Wang Y. Tang X. F. Wang S. M.. (2019). Metabolomics integrated with transcriptomics reveals redirection of the phenylpropanoids metabolic flux in ginkgo biloba. J. Agric. Food Chem. 67 (11), 3284–3291. doi: 10.1021/acs.jafc.8b06355 30802049

[B28] Murakami A. Ashida H. Terao J. (2008). Multitargeted cancer prevention by quercetin. Cancer Lett. 269 (2), 315–325. doi: 10.1016/j.canlet.2008.03.046 18467024

[B29] Ni Q. X. Wang Z. Q. Xu G. Z. Gao Q. X. Yang D. D. Morimatsu F.. (2013). Altitudinal variation of antioxidant components and capability in indocalamus latifolius (Keng) McClure leaf. J. Nutr. Sci. Vitaminol. 59 (4), 336–342. doi: 10.3177/jnsv.59.336 24064734

[B30] Pandey G. Khatoon S. Pandey M. M. Rawat A. K. S. (2018). Altitudinal variation of berberine, total phenolics and flavonoid content in thalictrum foliolosum and their correlation with antimicrobial and antioxidant activities. J. Ayurveda Integr. Med. 9 (3), 169–176. doi: 10.1016/j.jaim.2017.02.010 29102462 PMC6148047

[B31] Qiu J. (2007). Traditional medicine: a culture in the balance. Nature 448 (7150), 126–128. doi: 10.1038/448126a 17625539

[B32] Rathinasabapathi B. Fouad W. M. Sigua C. A. (2001). Beta-alanine betaine synthesis in the plumbaginaceae. purification and characterization of a trifunctional, s-adenosyl-L-methionine-dependent n-methyltransferase from limonium latifolium leaves. Plant Physiol. 126 (3), 1241–1249. doi: 10.1104/pp.126.3.1241 11457974 PMC116480

[B33] Rius S. P. Grotewold E. Casati P. (2012). Analysis of the P1 promoter in response to UV-b radiation in allelic variants of high-altitude maize. BMC Plant Biol. 12, 92. doi: 10.1186/1471-2229-12-92 22702356 PMC3489873

[B34] Senica M. Stampar F. Veberic R. Mikulic-Petkovsek M. (2017). The higher the better? differences in phenolics and cyanogenic glycosides in sambucus nigra leaves, flowers and berries from different altitudes. J. Sci. Food Agric. 97 (8), 2623–2632. doi: 10.1002/jsfa.8085 27734518

[B35] Setyawati A. Komariah Pujiasmanto B. Fatawi A. Batubara I. (2021). “Secondary metabolites of turmeric and ginger on various altitudes and soil characteristics,” in IOP Conference Series: Earth and Environmental Science, Vol. 724. doi: 10.1088/1755-1315/724/1/012020

[B36] Shang X. H. Huang D. Wang Y. Xiao L. Ming R. H. Zeng W. D.. (2021). Identification of nutritional ingredients and medicinal components of pueraria lobata and its varieties using UPLC-MS/MS-Based metabolomics. Molecules 26 (21), 6587. doi: 10.3390/molecules26216587 34770994 PMC8588241

[B37] Shan Z. J. Ye J. F. Hao D. C. Xiao P. G. Chen Z. D. Lu A. M. (2022). Distribution patterns and industry planning of commonly used traditional Chinese medicinal plants in China. Plant Divers. 44 (3), 255–261. doi: 10.1016/j.pld.2021.11.003 35769595 PMC9209863

[B38] Shukla S. Gupta S. (2010). Apigenin: A promising molecule for cancer prevention. Pharm. Res. 27 (6), 962–978. doi: 10.1007/s11095-010-0089-7 20306120 PMC2874462

[B39] Tripp E. A. Zhuang Y. B. Schreiber M. Stone H. Berardi A. E. (2018). Evolutionary and ecological drivers of plant flavonoids across a large latitudinal gradient. Mol. Phylogenet. Evol. 128, 147–161. doi: 10.1016/j.ympev.2018.07.004 30017824

[B40] Watanabe S. Matsumoto M. Hakomori Y. Takagi H. Shimada H. Sakamoto A. (2014). The purine metabolite allantoin enhances abiotic stress tolerance through synergistic activation of abscisic acid metabolism. Plant Cell Environ. 37 (4), 1022–1036. doi: 10.1111/pce.12218 24182190

[B41] Yang L. Yang C. Li C. Zhao Q. Liu L. Fang X.. (2016). Recent advances in biosynthesis of bioactive compounds in traditional Chinese medicinal plants. Sci. Bull. (Beijing) 61, 3–17. doi: 10.1007/s11434-015-0929-2 26844006 PMC4722072

[B42] Yariyama S. Ando S. Seo S. Nakaho K. Miyashita S. Kanayama Y.. (2019). Exogenous application of l-histidine suppresses bacterial diseases and enhances ethylene production in rice seedlings. Plant Pathol. 68 (6), 1072–1078. doi: 10.1111/ppa.13037

[B43] Zhang Y. Swart C. Alseekh S. Scossa F. Jiang L. Obata T.. (2018). The extra-pathway interactome of the TCA cycle: Expected and unexpected metabolic interactions. Plant Physiol. 177 (3), 966–979. doi: 10.1104/pp.17.01687 29794018 PMC6052981

[B44] Zhang S. Xin H. Q. Li Y. Zhang D. M. Shi J. Yang J. Z.. (2013). Skimmin, a coumarin from hydrangea paniculata, slows down the progression of membranous glomerulonephritis by anti-inflammatory effects and inhibiting immune complex deposition. Evidence-Based Complementary Altern. Med 2013, 819296. doi: 10.1155/2013/819296 PMC3748779 23990847

[B45] Zhang S. Yang J. Z. Li H. Y. Li Y. Liu Y. Zhang D. M.. (2012). Skimmin, a coumarin, suppresses the streptozotocin-induced diabetic nephropathy in wistar rats. Eur. J. Pharmacol. 692 (1-3), 78–83. doi: 10.1016/j.ejphar.2012.05.017 22664227

[B46] Zhao Y. (2012). Auxin biosynthesis: a simple two-step pathway converts tryptophan to indole-3-acetic acid in plants. Mol. Plant 5 (2), 334–338. doi: 10.1093/mp/ssr104 22155950 PMC3309920

[B47] Zhao W. Shi X. Li J. Guo W. Liu C. Chen X. (2014). Genetic, epigenetic, and HPLC fingerprint differentiation between natural and ex situ populations of rhodiola sachalinensis from changbai mountain, China. PloS One 9 (11), e112869. doi: 10.1371/journal.pone.0112869 25386983 PMC4227887

