# Supplementary material for: Pseudotargeted metabolomics revealed the adaptive mechanism of Draba oreades Schrenk at high altitude
Source: Front Plant Sci. 2022 Dec 8;13:1052640. doi: 10.3389/fpls.2022.1052640 (PMC9784223; doi:10.3389/fpls.2022.1052640)
Supplement: Supplementary file 1 [file DataSheet_1.docx]

**Pseudotargeted Metabolomics Revealed the Adaptive Mechanism of *Draba oreades* Schrenk at High altitude**

**Ling Lei^1✝^, Xuefeng Yuan^2✝^, Keyi Fu^2^, Yuan Chen^2^, Yijun Lu^1^, Na Shou^2^, Dandan Wu^2^, Xi Chen^2^, Jian Shi^4^, Minjuan Zhang^4^, Zhe Chen^3*^, and Zunji Shi^2*^**

^1^Clinical Psychology, Maternal and Child Health Hospital of Guangxi Zhuang Autonomous Region, Guangxi Key Laboratory of Reproductive Health and Birth Defect Prevention, Nanning, 530003, China

^2^State Key Laboratory of Herbage Improvement and Grassland Agro-ecosystems, Center for Grassland Microbiome, College of Pastoral Agriculture Science and Technology, Lanzhou University, Lanzhou, 730000, China

^3^Qinghai Normal University, Academy of Plateau Science and Sustainability, Xining, 810016, China

^4^Wuhan Metware Biotechnology Co., Ltd., Wuhan, 430075, China

**^✝^**Contributed equally

*Address correspondence to: Dr. Zhe Chen and Dr. Zunji Shi.

Qinghai Normal University, Academy of Plateau Science and Sustainability, Xining, 810016, China. Email: chenzhe@qhnu.edu.cn

State Key Laboratory of Herbage Improvement and Grassland Agro-ecosystems, Center for Grassland Microbiome, College of Pastoral Agriculture Science and Technology, Lanzhou University, Lanzhou, 730000, China. Email: shizj@lzu.edu.cn

**KEYWORDS: *Draba oreades* Schrenk; flavonoids; high altitude; machine learning; pseudotargeted metabolomics**

**Table S1** Physicochemical properties of soil in Gangshika Mountain.

| Altitude（m） | Spots | Water content (%) | Temperature (℃) | Salinity (mg/L) | conductivity (us/cm) |
| --- | --- | --- | --- | --- | --- |
| 4200 | H1 | 20.5 | 2.5 | 11 | 21 |
|  | H2 | 16.4 | 1.6 | 4 | 8 |
|  | H3 | 18.2 | 1.6 | 9 | 17 |
|  | H4 | 21.2 | 0.5 | 6 | 12 |
|  | H5 | 15.4 | 0.5 | 3 | 6 |
|  | H6 | 24.0 | 1.0 | 10 | 19 |
|  | H7 | 14.1 | 4.1 | 3 | 7 |
|  | H8 | 14.0 | 3.1 | 2 | 4 |
| 4000 | M1 | 19.2 | 9.2 | 6 | 12 |
|  | M2 | 21.4 | 6.2 | 8 | 16 |
|  | M3 | 21 | 6.8 | 10 | 19 |
|  | M4 | 22.7 | 6.3 | 10 | 19 |
|  | M5 | 19.9 | 8.9 | 9 | 16 |
|  | M6 | 15.9 | 7.9 | 6 | 11 |
|  | M7 | 21 | 6.2 | 15 | 28 |
|  | M8 | 17.3 | 8.1 | 9 | 17 |
| 3800 | L1 | 52.9 | 7.8 | 66 | 121 |
|  | L2 | 49.8 | 6 | 73 | 133 |
|  | L3 | 49.8 | 6 | 79 | 144 |
|  | L4 | 46.5 | 6.9 | 83 | 151 |
|  | L5 | 27.2 | 6.2 | 29 | 53 |
|  | L6 | 48.5 | 6.2 | 46 | 85 |
|  | L7 | 21.4 | 7.1 | 18 | 33 |
|  | L8 | 20.0 | 7.9 | 10 | 19 |

**Table S2** Total ultraviolet intensity of plant growing season in Gangshika Mountain from May to September.

| Groups | Altitude (m) | Total UV intensity (MJ/m^2^) |
| --- | --- | --- |
| Low-altitude | 3800 | 169.274 |
| Mid-altitude | 4000 | 172.660 |
| High-altitude | 4200 | 178.313 |


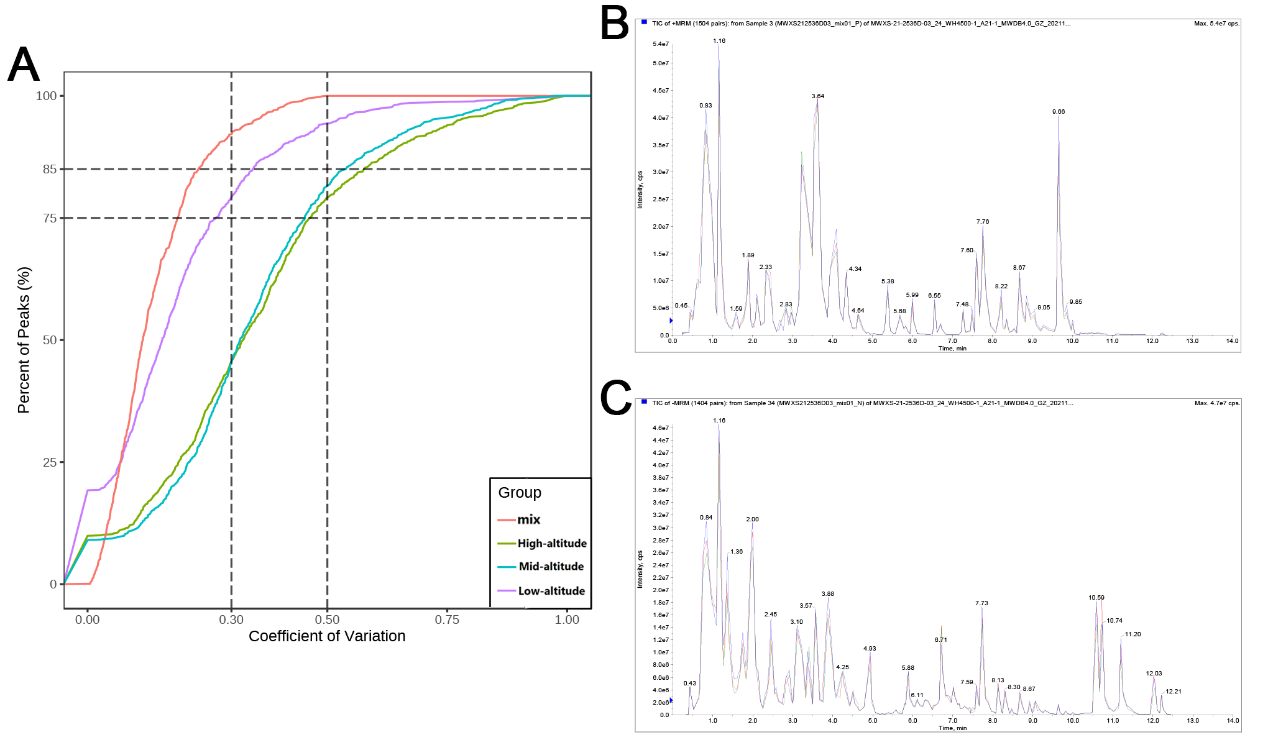


**Figure S1** Quality control (QC) charts of pseudotargeted metabolomics data. (A) CV distribution of samples. Red line represented the mix sample, which was used for QC. (B) TIC overlap diagram in positive ion mode. (C) TIC overlap diagram in negative ion mode.


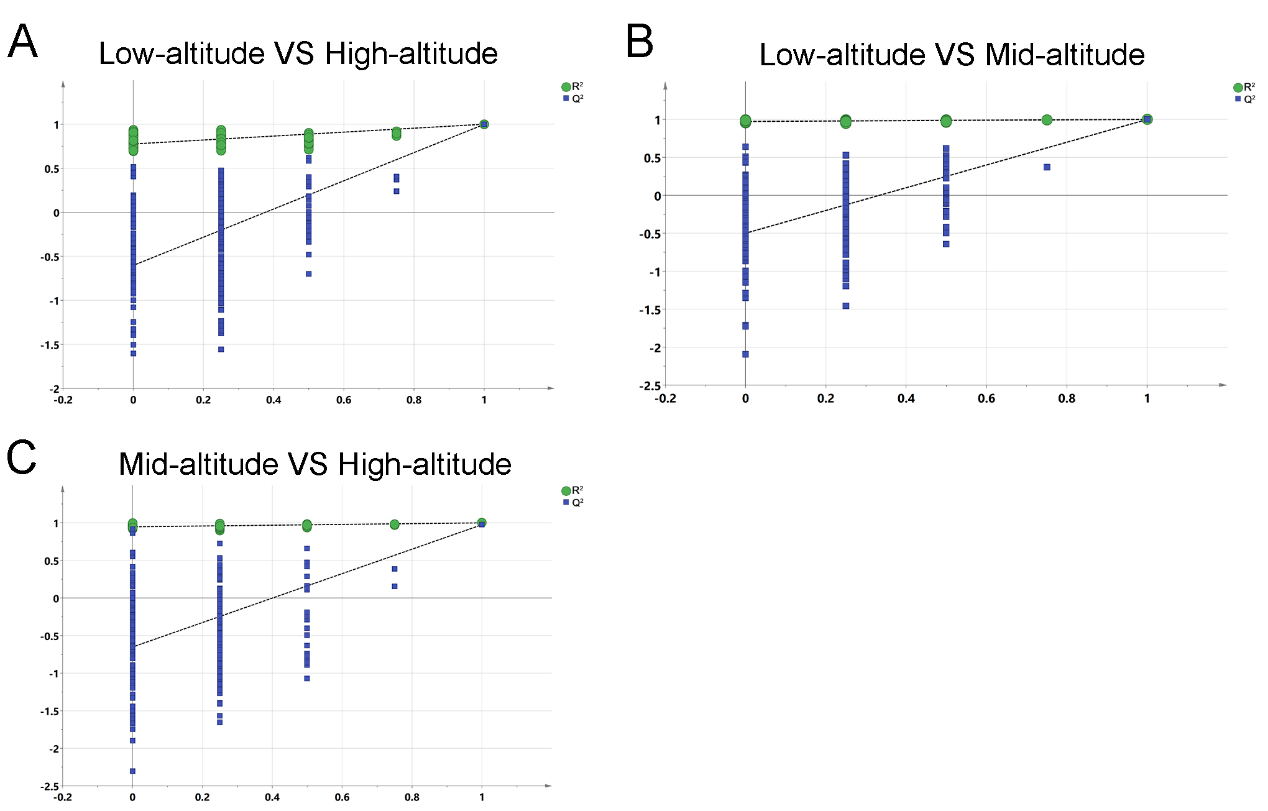


**Figure S2** Permutation plots obtained from OPLS-DA score plot based on 200 permutation tests. (A) Low-altitude vs High-altitude. (B) Low-altitude vs Mid-altitude. (C) Mid-altitude vs High-altitude. Green and blue dots represented R^2^ and Q^2^ of the model after Y was replaced. R^2^ and Q^2^ respectively represented the interpretation rate of the model to the matrix and the prediction ability of the model.
